# The first session is the one that counts: An exploratory study of therapeutic alliance

**DOI:** 10.3389/fpsyg.2022.1016963

**Published:** 2022-11-15

**Authors:** Francisco Javier del Río Olvera, Álvaro Rodríguez-Mora, Cristina Senín-Calderón, Juan F. Rodríguez-Testal

**Affiliations:** ^1^Department of Psychology, University of Cadiz, Cadiz, Spain; ^2^Institute of Biomedical Research and Innovation of Cádiz (INIBICA), University of Cadiz, Cadiz, Spain; ^3^Psychological Assistance Service, University of Cadiz, Cadiz, Spain; ^4^University Institute of Research for Sustainable Social Development (NDESS), University of Cadiz, Cadiz, Spain; ^5^Department of Personality, Evaluation and Psychological Treatment, University of Seville, Seville, Spain

**Keywords:** therapeutic alliance, working alliance, early alliance, STATIS DUAL, psychotherapy, common factors, ORS, SRS

## Abstract

**Background:**

The controversy about whether psychotherapy outcome is the consequence of the techniques themselves, common factors or both is still current. The importance of common factors has been demonstrated, although it is also known that they alone are insufficient. At the present time, the contextual model grants heavy weight to the therapeutic alliance in the first sessions and seems to predict positive final results. Furthermore, monitoring sessions has demonstrated that this alliance improves.

**Objectives:**

To analyze the relationship between the therapeutic alliance and patient’s perceived improvement during the first five sessions of therapy, and find out whether the therapeutic alliance is maintained or unstable within that timeframe.

**Methods:**

Thirty-four patients at a university psychological care service who had had at least five therapy sessions participated. Of these, 70.46% were women (*M*age = 24.24, *SD* = 6.73). The patients filled out the Outcome Rating Scale and Session Rating Scale the week before each session. Data were analyzed by the Dual STATIS method.

**Results:**

The compromise matrix explained 77.36% of the variability. The position of the vectors and the distribution of the position of the patients on the graph show that as their perception increased, therapeutic alliance remained stable. Moreover, the position of the vectors shows that the therapeutic alliance was forged in the first session and remained stable during the following sessions.

**Conclusion:**

This exploratory study demonstrated the importance of the first session in establishing the therapeutic alliance, and for it to remain stable, regardless of whether the rest of the therapeutic process has variations or changes. Novel use of the STATIS method for analyzing measurements in the first five sessions, showed that beginning the therapeutic intervention with a strong alliance, produced the favorable, lasting effects necessary for development of the intervention.

## Introduction

The therapeutic relationship is fundamental to any psychotherapeutic orientation and is one of the major factors contributing to the psychotherapy outcome (Baldwin et al., 2007; Finsrud et al., 2022). The effects and outcomes of psychotherapy are well known, however, whether these effects are due to specific factors related to treatment protocols, or to factors all psychotherapy modalities have in common, continues to be under discussion.

### Common factors vs. specific techniques

Psychotherapy is a complex multifactorial process, and it is quite likely that both common and specific factors have a role in the process leading to recovery. The debate on whether therapies work through common or specific mechanisms has kept up for decades (Cuijpers et al., 2019; Baier et al., 2020; Prochaska et al., 2020). This said, the only empirical conclusion that can be arrived at is that it is unknown whether therapies act through common or specific factors or both, or whether they interact (de Felice et al., 2019), and still more research is required to establish it (Cuijpers et al., 2019). The common factors are perhaps considered necessary, but clearly insufficient alone. The evidence suggests that these common factors must be considered therapeutic and must be given due attention in theory, research and practice (Wampold, 2015). The book *Persuasion and Healing: A Comparative Study of Psychotherapy* (Frank and Frank, 1993) describes four factors common to all psychotherapies considered functional elements. These elements are the relationship between patient and therapist, the theoretical basis that provides the therapy with credibility, certain therapist structural procedures or rituals, and a therapeutic context. The recently proposed contextual model has a particular common factor (Wampold, 2015) and includes three aspects fundamental to the therapeutic process: (a) the real relationship between patient and therapist, (b) the creation of expectations through the explanation of the disorder and treatment selected, and (c) starting up the specific therapeutic action of the therapy chosen for the patient’s problem. However, before these routes are activated, attention must be given the initial therapeutic relationship, the creation of a bond between the therapist and the patient (Wampold and Imel, 2015; Finsrud et al., 2022). Compared to the original model of common factors by Frank and Frank (1993), the contextual model of Wampold and Imel (2015) specifies and emphasizes even more the basic importance of the therapeutic alliance through the relationship established between the patient and the therapist.

The therapeutic alliance vs. the specific technique is considered by a large number of psychologists to be essential and predictive of a positive therapeutic outcome (Hogue et al., 2008; Stamoulos et al., 2016; Norcross and Wampold, 2018). The therapeutic relationship established at the beginning of therapy is considered valid for different measurement systems, types of intervention (including online therapy), patient characteristics, and consistency across countries (Flückiger et al., 2018). A recent review demonstrated the mediating role of the therapeutic alliance, especially its consolidation at treatment startup, in over 70% of the studies reviewed (Baier et al., 2020).

The therapeutic alliance is defined as a subjective experience and collaborative partnership between patient and therapist, according to the therapeutic goals, tasks or processes defined to achieve these goals, and with positive emotional bonds based on trust in a therapeutic context (Bordin, 1979; Horvath, 2018).

Along this line, it seems that common factors such as therapeutic alliance or empathy could be the most important elements for successful treatment, sometimes more than the active technical factors themselves, although these are still significant (Cook et al., 2010; Stamoulos et al., 2016). The complexity of analyzing the therapeutic alliance is demonstrated by therapist assessments, which are usually lower, and not necessarily congruent with those of the patient (Zilcha-Mano et al., 2016). However, these perspectives of therapeutic alliance by the patient and therapist could be influencing each other. Analysis of specific dyads in the relationship or bond between patient and therapist has shown that the congruence of this bond is related to better results and fewer symptoms in the following session (Rubel et al., 2018).

Therefore, the relationship between the therapeutic alliance and therapeutic outcome may be due to a reciprocal influence and not one-way (Flückiger et al., 2020a), especially at the beginning of the intervention. Patient characteristics, the initial distress levels, and therapist competence are equally important factors (Flückiger et al., 2020b).

### Monitoring procedure followed in this study

Routine assessment of the patient’s adherence to therapy is an important task, but requires strong resources (Waller and Turner, 2016). The clinician’s use of feedback tools to evaluate progress in psychotherapy sessions can help determine whether the therapeutic approach is effective or needs to be changed. These tools also provide an opportunity for patients to discuss their treatment and their own opinions of their progress. The tools and their scoring are transparent, which means that the therapist is not limited to collecting data for himself, but shares with the patient and together they interpret what the data show about the treatment (Janse et al., 2020).

Thus, Feedback-Informed Treatment (FIT) takes advantage of information collected during the sessions to determine when the patients run the most risk of dropping out (Coleman, 2018). Other procedures, such as Routine Outcome Monitoring (ROM), which focus on feedback on well-being and the therapeutic alliance, demonstrate improvements in the therapeutic alliance by enabling its analysis, repair and emotional regulation, insight and interpersonal learning (Brattland et al., 2019).

This kind of patient feedback enables clinical improvement of the therapeutic approach by measuring and modifying the treatment systematically based on common factors, such as improvement in the therapeutic alliance (Coleman, 2018). FIT involves frequent systematic evaluation of therapeutic progress indicators (measurement of results), and feedback on the therapeutic alliance (measurement of the process). The therapist creates a culture of requesting feedback from the patient and making changes based on that feedback (Prescott, 2017). The outcome is usually measured at the beginning of the session, checking the key elements of socio-emotional functioning to adapt and direct the session. Measurement of the process is usually at the end of the session, giving the patient the opportunity to give his opinion on the session and how well the critical elements of the therapeutic relations seem to be developing. This patient feedback is a step in the process; however, clinicians must be able to reflect on and act on this feedback (Schuckard et al., 2017).

Standardized tools can help professionals identify when the patients are not progressing in the therapy, and have been related with better results when the patient does not respond without them (Shimokawa et al., 2010). Along this line, The *American Psychology Association* (APA) Working Group on Evidence-Based Practice (American Psychological Association, 2006) recommends that clinicians use instruments that routinely evaluate treatment progress. Monitoring or following up on patient progress is an important tool for the therapist, since it helps base treatment on solid evidence. The use of instruments for following the process is also more and more frequent among therapists. The growing support of research for routine outcome monitoring has led to the design of monitoring procedures (Overington and Ionita, 2012). Among them are the *Partners for Change Outcome Management System* (PCOMS; Miller et al., 2005; Duncan and Reese, 2015), which arose from clinical practice and was designed for such real settings (Gimeno-Peón et al., 2019). The PCOMS employs two scales, the *Outcome Rating Scale* (ORS; Miller et al., 2003), focused on the results, and the *Session Rating Scale* (SRS; Duncan et al., 2003), which evaluates the therapeutic alliance in each session. Both scales have normative, reliability, and validity data comparable to those of the original American versions (Andrade-González et al., 2021). The PCOMS directly involves clinicians and patients in a continuous process of measurement and discussion of both progress and the therapeutic alliance, and is the first system to do so (Gimeno-Peón et al., 2019). PCOMS seems to improve the results of cognitive behavioral treatment (Duncan and Reese, 2015; Waller and Turner, 2016), reducing the number of sessions required for sufficient or expected improvement (Janse et al., 2017), improves symptoms in fewer sessions (Janse et al., 2017; Koementas-de Vos et al., 2018; Janse et al., 2020) and reduces dropout rates (Janse et al., 2020; de Jong et al., 2021). As the first three-to-five sessions are considered essential in establishing the therapeutic alliance (Flückiger et al., 2018, 2020b) and its counterpart, therapeutic dropout (Roos and Werbart, 2013; Cooper et al., 2018), we proposed an exploratory analysis of what happens in the therapeutic alliance in a period of time limited to the first five sessions, regardless of the changes that could take place during the rest of the therapeutic process. Thus, from a state perspective, which enables the changes in perception of the alliance and well-being/symptoms during the treatment to be analyzed (Zilcha-Mano et al., 2016), this study posed the following objectives: (1) analyze the relationship between therapeutic alliance and patient perceived improvement during the first five sessions of therapy, and (2) check whether the therapeutic alliance remains stable over time or varies, as well as its relationship with perceived improvement. We predicted that better therapeutic alliance (higher scores on the SRS) and satisfaction with therapy (high scores on the ORS) would be related to higher patient perception of wellbeing. We also estimated that as the first five therapy sessions progressed, the therapeutic alliance would become more robust (higher, more stable scores on the SRS) and this would have repercussions on the patient’s own perceived improvement.

## Materials and methods

### Participants

The sample was made up of 34 patients at a university psychological care service (university in southern Spain). The problems for which patients sought consultation fell within the following general diagnostic categories following the DSM-5 classification (American Psychological Association, 2006): anxiety disorders (*n* = 16), mood disorders (*n* = 7), personality disorders (*n* = 3), obsessive–compulsive spectrum and related disorders (*n* = 2), eating disorders (*n* = 1), somatic symptom and related disorders (*n* = 1), trauma and stress disorders (*n* = 3), and other clinical problems (*n* = 1). Women made up 70.6% of the patients. The mean age was 24.24 years (*SD* = 6.73, range 18 to 55), and 29.4% were in university degree programs related to the social sciences, 26.5% to health sciences, 20.6% arts and humanities, 11.8% sciences, and 11.8% engineering and technology. 52.9% were in fourth year, 14.7% first year, 11.8% in second year and 11.8% third year, and 7.8% were postgraduate students. This study was performed following the principles of the World Medical Association Declaration of Helsinki (64th General Assembly, October 2013), the International Conference on Harmonization Guidelines for Good Clinical Practice (September 1997), and the legislation of the country where it was carried out. All participants were informed of the objectives of the study and signed their informed consent for participating.

### Instruments

Original datasheet for sociodemographic and clinical data. This self-report questionnaire was prepared by the authors to collect sociodemographic, academic, and clinical data of the patients who came to the Psychological Care Service. The clinical data included questions about physiological, emotional, cognitive, and motor symptoms. Problems in interpersonal, academic, and vocational relations were also evaluated. The number and severity of the symptoms were used as the selection criteria for care in the Psychological Care Service. Less severe patients were referred to prevention workshops or group therapy for emotional problems.

Routine Outcome Monitoring (ROM) focused on feedback on wellbeing and the therapeutic alliance using standardized measures. As our objective was to analyze the relationship of the therapeutic alliance to the patient’s perceived well-being, two of the measures most commonly used and studied, the Outcome Rating Scale (ORS) and the Session Rating Scale (SRS; Prescott, 2017), were chosen from among the large number of potential measures for use in this study.

**Outcome Rating Scale** (ORS; Miller et al., 2003). This visual analog scale has four items that evaluate the person’s perception of his well-being, interpersonal functioning (family and intimate relationships), social functioning (work, school, and friends), and general well-being. The scale is administered in the time between sessions and evaluates therapeutic progress compared to the starting point. The authors of the scale found adequate overall internal consistency (Cronbach’s alpha = 0.93) and moderately strong correlations with the Outcome Questionnaire 45.2 (OQ-45.2; Lambert et al., 1996), suggesting signs of concurrent validity. The measure discriminated between non-clinical and clinical populations and in the clinical population, reflected high sensitivity to change after therapy. Excellent internal consistency and evidence of concurrent and discriminant validity were found in the validation of the measure with Spanish patients. The scale was highly sensitive to change (Andrade-González et al., 2021). With the sample in this study, internal consistency of the scale was 0.987.

**Session Rating Scale** (SRS; Duncan et al., 2003). This is a four-item measure in a visual analog scale format which evaluates how the person feels about the therapeutic alliance, achievement of the therapeutic objectives, the therapeutic approach and satisfaction with the therapy session in general. This scale evaluates the patient’s progress session by session and has been shown to be a useful measure of therapy effectiveness. A high score indicates a good therapeutic relationship. The Cronbach’s alpha found by the scale’s authors was 0.88, reflecting internal consistency. Test–retest reliability was r = 0.64. The SRS scale indicated concurrent validity with moderate correlations with the Helping Alliance Questionnaire II (HAQ-II; Luborsky et al., 1996). The Spanish validation had high internal consistency, measurement stability, evidences of convergent and discriminant validity, and adequate predictive validity (Andrade-González et al., 2021). Internal consistency of the scale found with study sample was 0.973.

### Procedure

Before starting therapeutic intervention, all the patients received information in writing on the possibility that their data could be used for research, protecting their personal information, and they signed their informed consent. At the end of the session, each patient was sent the ORS and SRS scales by email and they were asked to return them filled in by return email before the following session. This study analyzed the data of 34 patients who filled in at least five ORS and SRS evaluations. Among the sample inclusion criteria were to be enrolled as a student at the university where the service was located, be 18 years of age or older and be Spanish speakers. Participants who did not attend at least five sessions or did not fill in the scales were excluded.

The intervention sessions were carried out by Service therapists. After requesting attention at the Service, the patients were assigned to a single therapist. All of them were psychologists with a cognitive-behavioral orientation. The session contents were organized freely by each therapist depending on the therapeutic objectives resulting from the evaluation, and therefore, did not follow a systematized protocol. The therapists had an average clinical experience of 11.5 years (*SD* = 8.53).

### Statistical analysis

First, (dis)similarity of the data and the correlation coefficients between the different moments in time (RV) were found by Euclidean representation. For the compromise matrix, the weights at each moment in time and the percentage of variability explained on each of the axes were calculated. Finally, the interstructure analysis and STATIS-Dual graphs were prepared. All the calculations were done using the MultBiplot program developed by Vicente-Villardón (2015) at the University of Salamanca (Spain) Statistics Dept.

## Results

**Figure 1:**
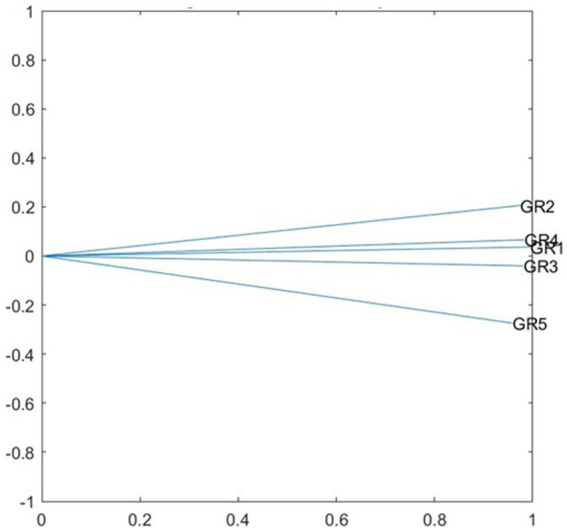
STATIS DUAL, interstructure analysis.

To be able to respond to the objectives set, work began by verifying the suitability of the data for multidimensional analysis. First the Euclidean projection of the data analyzed was presented (Figure 1). Each vector represents a moment in time of patient analysis, that is, Vector 1 represents the patient’s answers to the questionnaires after the first therapy session, Vector 2 after the second session, and so forth. The position in the Euclidean space is interpreted as the (dis)similarity between the different points in time analyzed. Vectors 1, 3, and 4 are closer and Vectors 2 and 5 slightly further apart. This interstructure plot shows the close relationship between the different points in time, and therefore, pertinence of the multidimensional analysis of the multiple tables, as well as the stability of the therapeutic alliance during the sessions. This close relationship between the different points in time is also observed in the Pearson correlation coefficients provided by the program as shown in Table 1. All the coefficients are over 0.9, except the one relating Vectors 2 and 5 (0.882), which are the furthest from the others in the plot.

**Table 1:** Pearson correlation coefficients between the five points of time.

	GR1	GR2	GR3	GR4
GR2	0.975			
GR3	0.970	0.947		
GR4	0.975	0.964	0.941	
GR5	0.943	0.882	0.940	0.928

GR corresponds to the time point, from 1 to 5. In all cases, p < 0.01.

The weight assigned to each of the original matrices (points in time) in the resulting compromise matrix should also be mentioned. Matrix 1 had a pondered weight of 0.203, Matrix 2 0.199, Matrix 3 0.201, Matrix 4 0.201, and Matrix 5 0.196. These data suggest that the most important contact in the therapy sessions is during the first one, because it is the one with the highest weight. At the same time, there is no great difference between the weights found at each point in time, which shows that the compromise matrix is adequate for the analyses. The two axes of the compromise matrix explain a total of 77.36% of the variability (Axis 1 42.75%, and Axis 2 34.61%).

**Figure 2:**
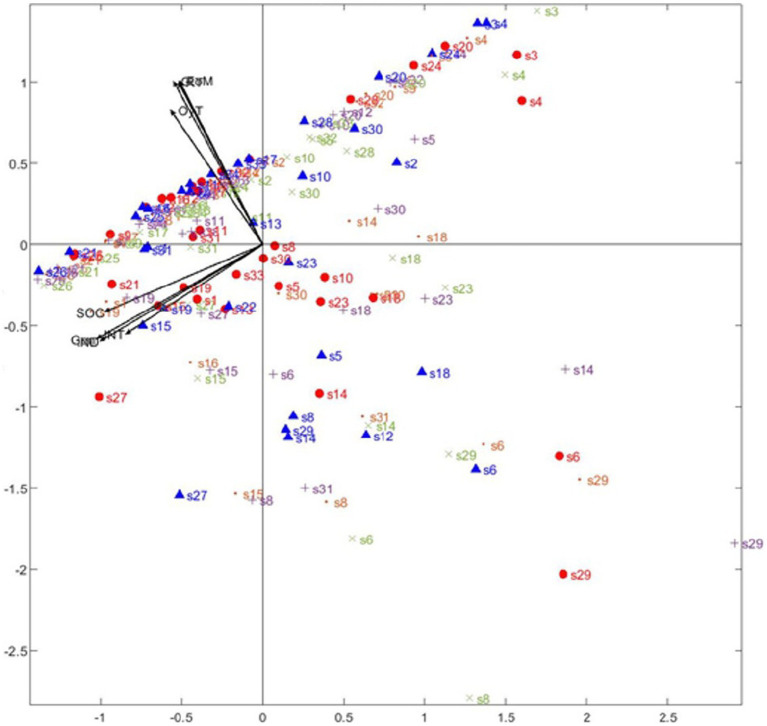
STATIS Dual Analysis. Vectors in the second quadrant: RT (Relationship with therapist), EoM (Approach or Method), OyT (Goals and Topics), and G (Overall alliance). Vectors in the third quadrant: IND (Individual well-being). INT (interpersonal functioning with family, and intimate relationships), SOC (satisfaction with work/school and relationships outside of home) and Gen (overall well-being).

Figure 2, which presents the projection of the participants in STATIS Dual, was analyzed to respond to Objective 1 on any possible relationship between the therapeutic alliance and patients’ perceived improvement. The contribution of each of the measurements was differentiated by clustering the results of the SRS questionnaire (therapeutic alliance) and the ORS questionnaire (satisfaction) in vector bundles. Session 1 is represented by a triangle, Session 2 by a circle, Session 3 by an x, Session 4 by a +, and Session 5 by a dot, and the numbers represent the 34 participants in the study. Most of the points are distributed parallel to the ORS scale vectors (perceived personal well-being) and perpendicular to the vectors on the SRS scale (therapeutic alliance). It should be emphasized that most of the points cut the SRS scale vectors in half, and as this is the length of the vector of the total patient mean scores, the score on this scale is high (good therapeutic relationship), and is only lowered by those participants represented on Plane 4. Similarly, most of the participant scores appear parallel to the ORS scale vectors, where the sessions with the most disperse scores are the first (triangle) and the second (circle), and the rest are mostly grouped around the 0 point of the scale. Therefore, starting after the second session, patient perception of themselves improves. The information provided by the combination of the two vectors and the position of the patients shows that as their perception improves, the score on the therapeutic alliance remains high.

For the second objective, about whether the therapeutic alliance remains stable over time, the relationship between the vectors and the patient scores in Figure 2 was analyzed. The vectors cluster in two well-differentiated groups, corresponding to the results on each of the questionnaires. The RT (Relationship with the Therapist), EoM (Approach or Method) and OyT (Objectives and Themes), and G (General) vectors from the Session Evaluation Scale (SRS), which defines the relationship with the therapist and the therapeutic session which was just completed, are all in the second quadrant. In the third quadrant are the IND (individual personal wellbeing) INT (interpersonal functioning with family and close relatives), SOC (social functioning at work, study and with friends) and Gen (General feeling of well-being), on the Outcome Rating Scale (ORS), in which the patients evaluate their own progress and well-being. This is statistically coherent, as the vectors are clustered by questionnaires used, showing high correlation between questionnaire scales. The relationship between the two questionnaires by the degree of the angles formed by the groups of vectors also has to be interpreted. In this case, there is almost a 90-degree angle, which shows that the groups are statistically independent. This may be interpreted as absence of any relationship between them, such that trust in the therapist is formed at the beginning of the sessions and does not vary over the five sessions the measurement was made in, regardless of any improvement the patient may feel. At the same time, the perpendicular relationship of the subjects to the SRS scale shows that once the therapeutic alliance is formed at the beginning of the sessions, it remains stable throughout.

## Discussion

The debate about the role of the therapeutic alliance remains open, showing how complex the study of this process is and the need to determine what other factors contribute, and to what extent, to the functioning of psychological therapy (Cuijpers et al., 2019; Moriana et al., 2022). This difficulty in finding precisely even whether the therapeutic alliance is a process common to the different therapeutic procedures, or whether it is rather a specific factor (Baier et al., 2020), demonstrates that we are still only at the beginning of scientific knowledge of the therapeutic process (Onken et al., 2014), perhaps still with basic analyses that are really additive, linear and less directed at identifying patterns of change (de Felice et al., 2019).

However, as the therapeutic alliance is a necessary condition of therapy, predictive not only of the outcome of intervention (Norcross and Wampold, 2018; Flückiger et al., 2020a; Finsrud et al., 2022), but also of dropping out (Cooper et al., 2018), and the first sessions when the patient-therapist alliance is established and stabilized, are important (Flückiger et al., 2018, 2020a; Del Re et al., 2021), the objectives posed for this study were to analyze the relationship between the therapeutic alliance and perceived improvement during the first five sessions of the intervention, and check whether the therapeutic alliance remained stable during this period. For the first objective, and as a novelty in the analysis of the variables intervening in therapy, it should be mentioned that the STATIS procedure employed in this study clearly differentiated the contribution of each of the measurements, by clustering the results of the SRS questionnaire (therapeutic alliance), and the ORS questionnaire (satisfaction), in different bundles of vectors. This made it possible to analyze how the therapeutic alliance influences patient perception of their evolution in therapy. As mentioned under Results, when the questionnaire vectors are at 90-degree angles to each other, they are independent of each other.

Figure 2 also shows that the most inconsistent participant scores are those corresponding to Sessions 1 and 2, and the rest of the scores cluster mostly above the 0 point on the ORS scale, illustrating the improvement in patients as they progress during this five-session time period. Therefore, the first sessions are essential in forging the therapeutic alliance. However, the novel finding of this study is that while the reciprocal relationship between the therapeutic alliance and symptoms/distress described in the literature, in which the symptoms predict the alliance and the alliance predicts the symptoms (Flückiger et al., 2020a), the therapeutic alliance may remain more stable, or be constituted on a basis within this margin of time, but from the first session. Doubtless there are many more details forming part of these key moments in the therapeutic relationship, for example, the perception of agency during therapy, which would affect the relationship between the alliance and symptoms (Huber et al., 2021). The idea that the therapeutic alliance is an initially stable base, could be related precisely to the fact that it facilitates action of specific factors, like being receptive to Socratic dialog in cognitive therapy (Baier et al., 2020).

Continuing with the objectives set for this study, it should be mentioned that once the therapeutic alliance has been established, it remains stable throughout the first five sessions, since the relationship between the vectors of the therapeutic alliance and patient perceived improvement are independent. In fact, most of the patient scores are perpendicular to the SRS questionnaire vectors. Similarly, by weighting the results, the clinical session observed to be the most influential in forming the therapeutic alliance is the first.

The data therefore show that a good therapeutic alliance between therapist and patients is founded from the beginning of the relationship, and that this relationship remains stable throughout the first five sessions analyzed, even in spite of the patients’ perception of how they are nearing therapeutic goals up to that time. It is therefore possible that, if this base is already solid, repair of the therapeutic alliance would be more propitious, and there would be fewer of the interruptions and dropouts (Humer et al., 2021) characteristic of intervention. All in all, it is clear that other considerations not included in this study, such as the patient’s functional characteristics, mainly interpersonal problems, which are clearly determinant in shaping the therapeutic alliance with their own relational dimension (Zilcha-Mano and Errázuriz, 2017), may be participating in this process and at the moment in time described.

*A priori*, these results may seem to suggest that the therapeutic alliance does not influence patient improvement, but they do underline that beginning the therapeutic intervention with a strong alliance produces favorable effects in patient perception, and retains its influence during the sessions the observation was made. This result coincides with the consideration of the therapeutic alliance as a critical process at the beginning of treatment (Flückiger et al., 2020a), showing a certain pattern of alliance (Zilcha-Mano and Errázuriz, 2017), and at that time, less susceptible to alteration or rupture (Baier et al., 2020). Therefore, more than emphasizing variations in the therapeutic relationship, with sudden losses and gains (Zilcha-Mano et al., 2019), the results of this study demonstrate that in the first five sessions, there is a certain stability in the influence of the therapeutic alliance on the patient’s perceived improvement, and clearly, from the first session, at least in a cognitive-type intervention. However, the specificity of the therapeutic alliance itself may require more precision, for example in those therapeutic formats based on relational intervention, where most characteristics and the dynamics of the therapeutic alliance are identified (Baier et al., 2020).

Some studies have focused on the differences between therapeutic evaluation and evaluation of the patient. For example, Zilcha-Mano et al. (2019) observed that when therapists find ruptures, but not the patients, there is a gain in the following sessions. In this study, evaluation of the alliance by the patient was taken into account, and that would fit in with the observation of its early stabilization. Possibly, consideration of the therapist’s evaluation would show an association between the two evaluations in later sessions (Kivlighan et al., 2016).

The results of this study are exploratory and had some limitations that recommend their cautious interpretation. In the first place, the sample is small and may not be representative of the general clinical population, since all were university students who voluntarily requested help with emotional problems. This comment involves implicit consideration of the severity of the request for help, and therefore the results may not be generalizable outside of the university context, or to a more severe clinical population. The therapeutic alliance is known to be problematic from the beginning in personality disorders, especially with interpersonal problems (Schenk et al., 2021), or post-traumatic stress (McLaughlin et al., 2014), but it should also be observed whether there is an optimum or propitious moment for establishing the therapeutic alliance as the base, as suggested in this study. In particular, and since users with the mentioned diagnoses participated in this study, no particular difficulty was observed in the participants. In the second place, many of the dropouts appeared after the first session, so having considered the first five sessions already suggests that a favorable alliance with the therapist has been formed (Del Re et al., 2021). Thirdly, the study is based on the first five sessions following observations in the literature, but the result of the whole therapeutic process was not analyzed, excluding the possibility of checking whether the final result was favorable, or whether, definitely, the result was due to the therapeutic alliance base as described, whether the alliance had variations, or whether there were ruptures, since none of the patients were administered postintervention evaluation, and it was their subjective perception of well-being that was analyzed. To contextualize the intervention, on average, the duration of the intervention in this university service is about 8–10 sessions. Fourth, related to the above, the measures were self-reports, which could indicate differences in evaluation from an outside observer (Del Re et al., 2021). In regard to this point, it should be taken into account that participant answers were not anonymous, which could be a skew factor in the study results. Fifth, it should be mentioned that certain therapist variables were not controlled for (gender, years of experience, number and severity of cases usually dealt with, theoretical orientation, training, or supervision; Johns et al., 2019), and it is very important to know what each therapist can contribute (Del Re et al., 2021). In fact, one possible effect to be kept in mind is whether there could be differences in cases when therapists had only one patient assigned and when they had several cases at once. Certain patient variables were not controlled for either (age, gender, socioeconomic status, motivation for the change, pathology with and without history, medication, etc.). It should further be kept in mind that the measure used was a self-report evaluation and could therefore have been affected by social desirability. And finally, the time the patients took to turn in the test results before the next session was not controlled, so there could have been differences between those who filled in the tests immediately after the session and those who did so right before the next therapy session.

In brief, this research highlighted the importance of the therapeutic alliance, already established as a causal variable and/or mediator of therapeutic change (Baier et al., 2020; Crits-Christoph and Gibbons, 2021). Although it is exploratory, our study showed, not only that the first moments of the intervention are important (between the third and fifth session, according to the literature), but especially the first session, in which independence from the measure of well-being suggests the solidity of this necessary condition of the alliance for intervention. This study also contributes its novel analysis by STATIS, based on follow-up of the measures in each session and characterization of their relationship of (in)dependence. This methodological and statistical approach can facilitate the analysis, follow-up, and probably, in a practical context, negotiation of the therapeutic relationship (Brattland et al., 2019; Crits-Christoph and Gibbons, 2021). Although it underlines the base or stability of the therapeutic alliance in the first sessions, it should be emphasized that the therapeutic alliance is not in itself a stable process, it is an active process of change (Zilcha-Mano et al., 2019), a dynamic part of the dyadic patient-therapist relationship (Zilcha-Mano et al., 2016). As analytical models tend to be linear (de Felice et al., 2019), it should be stressed that this therapeutic alliance process is probably not, and therefore the importance of subjecting it to the type of analysis in this study with respect to rupture and repair of the therapeutic alliance during the course of the intervention (Baier et al., 2020). Finally, in later studies, this method would have to be tested for the analysis of intervening moderators, such as important effects of the therapists (Johns et al., 2019; Firth et al., 2020), characteristics of the patients (Baier et al., 2020), and different patterns of change (de Felice et al., 2019).

## Data availability statement

The raw data supporting the conclusions of this article will be made available by the authors, without undue reservation.

## Ethics statement

Ethical approval was not provided for this study on human participants because participants are patients of a university psychological care service who received free therapy and signed a written informed consent to allow their data to be used for research purposes. The patients/participants provided their written informed consent to participate in this study.

## Author contributions

All authors listed have made a substantial, direct, and intellectual contribution to the work and approved it for publication.

## Conflict of interest

The authors declare that the research was conducted in the absence of any commercial or financial relationships that could be construed as a potential conflict of interest.

## Publisher’s note

All claims expressed in this article are solely those of the authors and do not necessarily represent those of their affiliated organizations, or those of the publisher, the editors and the reviewers. Any product that may be evaluated in this article, or claim that may be made by its manufacturer, is not guaranteed or endorsed by the publisher.

## References

[ref1] American Psychological Association (2006). Evidence-based practice in psychology. Am. Psychol. 61, 271–285. doi: 10.1037/0003-066X.61.4.271 16719673

[ref2] Andrade-González N. Rodrigo-Holgado I. Fernández-Rozas J. Cáncer P. F. Lahera G. Fernández-Liria A.. (2021). Spanish versions of the outcome rating scale and the session rating scale: normative data, reliability, and validity. Front. Psychol. 12:663791. doi: 10.3389/fpsyg.2021.663791, PMID: 34484027 PMC8414252

[ref3] Baier A. L. Kline A. C. Feeny N. C. (2020). Therapeutic alliance as a mediator of change: a systematic review and evaluation of research. Clin. Psychol. Rev. 82:101921. doi: 10.1016/j.cpr.2020.101921, PMID: 33069096

[ref4] Baldwin S. A. Wampold B. E. Imel Z. E. (2007). Untangling the alliance-outcome correlation: exploring the relative importance of therapist and patient variability in the alliance. J. Consult. Clin. Psychol. 75, 842–852. doi: 10.1037/0022-006X.75.6.842, PMID: 18085902

[ref5] Bordin E. S. (1979). The generalizability of the psychoanalytic concept of the working alliance. Psychotherapy: theory. Research & Practice 16, 252–260. doi: 10.1037/h0085885

[ref6] Brattland H. Koksvik J. M. Burkeland O. Klöckner C. A. Lara-Cabrera M. L. Miller S. D.. (2019). Does the working alliance mediate the effect of routine outcome monitoring (ROM) and alliance feedback on psychotherapy outcomes? A secondary analysis from a randomized clinical trial. J. Couns. Psychol. 66, 234–246. doi: 10.1037/cou0000320, PMID: 30702322

[ref7] Coleman S. L. (2018). Common factors and common elements: use of data science-derived innovations to improve school-based counseling. Contemp School Psychol 22, 512–524. doi: 10.1007/s40688-018-0192-z

[ref8] Cook J. M. Biyanova T. Elhai J. Schnurr P. P. Coyne J. C. (2010). What do psychotherapists really do in practice? An internet study of over 2,000 practitioners. Psychotherapy: theory. Research, Practice, Training 47, 260–267. doi: 10.1037/a0019788, PMID: 22402052 PMC3676965

[ref9] Cooper A. A. Kline A. C. Baier A. L. Feeny N. C. (2018). Rethinking research on prediction and prevention of psychotherapy dropout: a mechanism-oriented approach. Behav. Modif. 0145445518792251. doi: 10.1177/0145445518792251 [Epub ahead of print]., PMID: 30079755

[ref10] Crits-Christoph P. Gibbons M. B. C. (2021). “Psychotherapy process–outcome research: advances in understanding causal connections,” in Bergin and Garfield’s handbook of psychotherapy and behavior change. eds. Barkham M. Lutz W. Castonguay L. G. (Hoboken, NJ: Wiley & Sons), 263–296.

[ref11] Cuijpers P. Reijnders M. Huibers M. J. H. (2019). The role of common factors in psychotherapy outcomes. Annu. Rev. Clin. Psychol. 15, 207–231. doi: 10.1146/annurev-clinpsy-050718-095424 30550721

[ref12] de Felice G. Giuliani A. Halfon S. Andreassi S. Paoloni G. Orsucci F. F. (2019). The misleading dodo bird verdict. How much of the outcome variance is explained by common and specific factors? New Ideas Psychol. 54, 50–55. doi: 10.1016/j.newideapsych.2019.01.006

[ref13] de Jong K. Conijn J. M. Gallagher R. A. V. Reshetnikova A. S. Heij M. Lutz M. C. (2021). Using progress feedback to improve outcomes and reduce drop-out, treatment duration, and deterioration: a multilevel meta-analysis. Clin. Psychol. Rev. 85:102002. doi: 10.1016/j.cpr.2021.102002, PMID: 33721605

[ref14] Del Re A. C. Flückiger C. Horvath A. O. Wampold B. E. (2021). Examining therapist effects in the alliance–outcome relationship: a multilevel meta-analysis. J. Consult. Clin. Psychol. 89, 371–378. doi: 10.1037/ccp0000637, PMID: 33829817

[ref15] Duncan B. L. Miller S. D. Sparks J. A. Claud D. A. Reynolds L. R. Bronw J.. (2003). The session rating scale: preliminary psychometric properties of a “working” alliance measure. J. Brief Therapy 3, 3–12.

[ref16] Duncan B. L. Reese R. J. (2015). The Partners for Change Outcome Management System (PCOMS) revisiting the client’s frame of reference. Psychotherapy 52, 391–401. doi: 10.1037/pst0000026, PMID: 26641369

[ref17] Finsrud I. Nissen-Lie H. A. Vrabel K. Høstmælingen A. Wampold B. E. Ulvenes P. G. (2022). It’s the therapist and the treatment: the structure of common therapeutic relationship factors. Psychother. Res. 32, 139–150. doi: 10.1080/10503307.2021.1916640, PMID: 33938407

[ref18] Firth N. Saxon D. Stiles W. B. Barkham M. (2020). Therapist effects vary significantly across psychological treatment care sectors. Clin. Psychol. Psychother. 27, 770–778. doi: 10.1002/cpp.2461, PMID: 32307805

[ref19] Flückiger C. Del Re A. C. Wampold B. E. Horvath A. O. (2018). The alliance in adult psychotherapy: a meta-analytic synthesis. Psychotherapy 55, 316–340. doi: 10.1037/pst0000172, PMID: 29792475

[ref20] Flückiger C. Del Re A. C. Wlodasch D. Horvath A. O. Solomonov N. Wampold B. E. (2020b). Assessing the alliance–outcome association adjusted for patient characteristics and treatment processes: a meta-analytic summary of direct comparisons. J. Couns. Psychol. 67, 706–711. doi: 10.1037/cou0000424, PMID: 32212755 PMC7529648

[ref21] Flückiger C. Rubel J. Del Re A. C. Horvath A. O. Wampold B. E. Crits-Christoph P.. (2020a). The reciprocal relationship between alliance and early treatment symptoms: a two-stage individual participant data meta-analysis. J. Consult. Clin. Psychol. 88, 829–843. doi: 10.1037/ccp0000594, PMID: 32757587

[ref22] Frank J. D. Frank J. B. (1993). Persuasion and healing: A comparative study of psychotherapy. Baltimore, Maryland, USA: JHU Press.

[ref23] Gimeno-Peón A. Prado-Abril J. Inchausti F. Barrio-Nespereira A. Álvarez-Casariego M. T. Duncan B. L. (2019). Systematic client feedback: a naturalistic pilot study. Ansiedad y Estrés 25, 132–137. doi: 10.1016/j.anyes.2019.04.005

[ref24] Hogue A. Henderson C. E. Dauber S. Barajas P. C. Fried A. Liddle H. A. (2008). Treatment adherence, competence, and outcome in individual and family therapy for adolescent behavior problems. J. Consult. Clin. Psychol. 76, 544–555. doi: 10.1037/0022-006X.76.4.544, PMID: 18665684 PMC2843085

[ref25] Horvath A. O. (2018). Research on the alliance: knowledge in search of a theory. Psychother. Res. 28, 499–516. doi: 10.1080/10503307.2017.1373204, PMID: 28899230

[ref26] Huber J. Jennissen S. Nikendei C. Schauenburg H. Dinger U. (2021). Agency and alliance as change factors in psychotherapy. J. Consult. Clin. Psychol. 89, 214–226. doi: 10.1037/ccp0000628, PMID: 33829809

[ref27] Humer E. Schramm E. Klein J. P. Härter M. Hautzinger M. Pieh C.. (2021). Effects of alliance ruptures and repairs on outcomes. Psychother. Res. 31, 977–987. doi: 10.1080/10503307.2021.1874070, PMID: 33455531

[ref28] Janse P. D. De Jong K. Van Dijk M. K. Hutschemaekers G. J. M. Verbraak M. J. P. M. (2017). Improving the efficiency of cognitive-behavioural therapy by using formal client feedback. Psychother. Res. 27, 525–538. doi: 10.1080/10503307.2016.1152408, PMID: 27013204

[ref29] Janse P. D. de Jong K. Veerkamp C. van Dijk M. K. Hutschemaekers G. J. M. Verbraak M. J. P. M. (2020). The effect of feedback-informed cognitive behavioral therapy on treatment outcome: a randomized controlled trial. J. Consult. Clin. Psychol. 88, 818–828. doi: 10.1037/ccp0000549, PMID: 32658496

[ref30] Johns R. G. Barkham M. Kellett S. Saxon D. (2019). A systematic review of therapist effects: a critical narrative update and refinement to review. Clin. Psychol. Rev. 67, 78–93. doi: 10.1016/j.cpr.2018.08.004, PMID: 30442478

[ref31] Kivlighan D. M. Hill C. E. Gelso C. J. Baumann E. (2016). Working alliance, real relationship, session quality, and client improvement in psychodynamic psychotherapy: a longitudinal actor partner interdependence model. J. Couns. Psychol. 63, 149–161. doi: 10.1037/cou0000134, PMID: 26689627

[ref32] Koementas-de Vos M. M. W. Nugter M. A. Engelsbel F. De Jong K. (2018). Does progress feedback enhance the outcome of group psychotherapy? Psychotherapy 55, 151–163. doi: 10.1037/pst0000164, PMID: 29863395

[ref33] Lambert M. J. Hansen N. B. Umphress V. Lunnen K. Okiishi J. Burlingame G. M.. (1996). Administration and scoring manual for the outcome questionnaire (OQ-45.1). United States: American Professional Credentialing Services.

[ref34] Luborsky L. Barber J. P. Siqueland L. Johnson S. Najavits L. M. Frank A.. (1996). The revised helping Alliance questionnaire (HAq-II): psychometric properties. J. Psychother. Pract. Res. 5, 260–271. 22700294 PMC3330423

[ref35] McLaughlin A. A. Keller S. M. Feeny N. C. Youngstrom E. A. Zoellner L. A. (2014). Patterns of therapeutic alliance: rupture–repair episodes in prolonged exposure for posttraumatic stress disorder. J. Consult. Clin. Psychol. 82, 112–121. doi: 10.1037/a0034696, PMID: 24188510 PMC3921691

[ref36] Miller S. D. Brown J. Sparks J. A. Claud D. A. (2003). The outcome rating scale: a preliminary study of the reliability, validity, and feasibility of a brief visual analog measure. Ournal of brief. Therapy 2, 91–100.

[ref37] Miller S. D. Duncan B. L. Sorrell R. Brown G. S. (2005). The partners for change outcome management system. J. Clin. Psychol. 61, 199–208. doi: 10.1002/jclp.20111 15609362

[ref38] Moriana J. A. Corpas J. Gálvez-Lara M. (2022). Towards a consensus in the evaluation of the evidence of psychological treatments. Clínica y Salud 33, 91–92. doi: 10.5093/clysa2022a9

[ref39] Norcross J. C. Wampold B. E. (2018). A new therapy for each patient: evidence-based relationships and responsiveness. J. Clin. Psychol. 74, 1889–1906. doi: 10.1002/jclp.22678, PMID: 30334258

[ref40] Onken L. S. Carroll K. M. Shoham V. Cuthbert B. N. Riddle M. (2014). Reenvisioning clinical science: unifying the discipline to improve the public health. Clin. Psychol. Sci. 2, 22–34. doi: 10.1177/2167702613497932, PMID: 25821658 PMC4374633

[ref41] Overington L. Ionita G. (2012). Progress monitoring measures: a brief guide. Canadian Psychology / Psychologie canadienne 53, 82–92. doi: 10.1037/a0028017

[ref42] Prescott D. S. (2017). “Feedback-informed treatment: an overview of the basics and core competencies,” in Feedback-informed treatment in clinical practice: Reaching for excellence. eds. Prescott D. S. Maeschalck C. L. Miller S. D. (Washington: American Psychological Association), 37–52.

[ref43] Prochaska J. O. Norcross J. C. Saul S. F. (2020). Generating psychotherapy breakthroughs: Transtheoretical strategies from population health psychology. Am. Psychol. 75, 996–1010. doi: 10.1037/amp0000568, PMID: 31763861

[ref44] Roos J. Werbart A. (2013). Therapist and relationship factors influencing dropout from individual psychotherapy: a literature review. Psychother. Res. 23, 394–418. doi: 10.1080/10503307.2013.775528, PMID: 23461273

[ref45] Rubel J. A. Bar-Kalifa E. Atzil-Slonim D. Schmidt S. Lutz W. (2018). Congruence of therapeutic bond perceptions and its relation to treatment outcome: within- and between-dyad effects. J. Consult. Clin. Psychol. 86, 341–353. doi: 10.1037/ccp0000280, PMID: 29389143

[ref46] Schenk N. Fürer L. Zimmermann R. Steppan M. Schmeck K. (2021). Alliance ruptures and resolutions in personality disorders. Curr. Psychiatry Rep. 23:1. doi: 10.1007/s11920-020-01212-w, PMID: 33305340 PMC7728649

[ref47] Schuckard E. Miller S. D. Hubble M. A. (2017). “Feedback-informed treatment: historical and empirical foundations,” in Feedback-informed treatment in clinical practice: Reaching for excellence. eds. Prescott D. S. Maeschalck C. L. Miller S. D. (Washington: American Psychological Association), 13–35.

[ref48] Shimokawa K. Lambert M. J. Smart D. W. (2010). Enhancing treatment outcome of patients at risk of treatment failure: meta-analytic and mega-analytic review of a psychotherapy quality assurance system. J. Consult. Clin. Psychol. 78, 298–311. doi: 10.1037/a0019247, PMID: 20515206

[ref49] Stamoulos C. Trepanier L. Bourkas S. Bradley S. Stelmaszczyk K. Schwartzman D.. (2016). Psychologists’ perceptions of the importance of common factors in psychotherapy for successful treatment outcomes. J. Psychother. Integr. 26, 300–317. doi: 10.1037/a0040426

[ref50] Vicente-Villardón J. L. (2015). MULTBIPLOT: a package for multivariate analysis using Biplots. Available at: http://biplot.usal.es/ClassicalBiplot/index.html

[ref51] Waller G. Turner H. (2016). Therapist drift redux: why well-meaning clinicians fail to deliver evidence-based therapy, and how to get back on track. Behav. Res. Ther. 77, 129–137. doi: 10.1016/j.brat.2015.12.005, PMID: 26752326

[ref52] Wampold B. E. (2015). How important are the common factors in psychotherapy? An update. World Psychiatry 14, 270–277. doi: 10.1002/wps.20238, PMID: 26407772 PMC4592639

[ref53] Wampold B. E. Imel Z. E. (2015). The great psychotherapy debate: The evidence for what makes psychotherapy work. 2nd Edn. London: Routledge.

[ref54] Zilcha-Mano S. Errázuriz P. (2017). Early development of mechanisms of change as a predictor of subsequent change and treatment outcome: the case of working alliance. J. Consult. Clin. Psychol. 85, 508–520. doi: 10.1037/ccp0000192, PMID: 28345940

[ref55] Zilcha-Mano S. Eubanks C. F. Muran J. C. (2019). Sudden gains in the alliance in cognitive behavioral therapy versus brief relational therapy. J. Consult. Clin. Psychol. 87, 501–509. doi: 10.1037/ccp0000397, PMID: 31008637 PMC6533161

[ref56] Zilcha-Mano S. Muran J. C. Hungr C. Eubanks C. F. Safran J. D. Winston A. (2016). The relationship between alliance and outcome: analysis of a two-person perspective on alliance and session outcome. J. Consult. Clin. Psychol. 84, 484–496. doi: 10.1037/ccp0000058, PMID: 27054824 PMC4873423

